# The Role of Chloride Ions in Serotonin Transport

**DOI:** 10.1101/2025.05.20.654092

**Published:** 2025-05-20

**Authors:** Jiahui Huang, Annika Backer, Stacy Uchendu, Bethlehem Bekele, Qingyang Chen, Esam Orabi, Robyn Stix, Yuan-Wei Zhang, Gary Rudnick, Eva Hellsberg, Lucy R. Forrest

**Affiliations:** 1 Computational Structural Biology Section, National Institutes of Neurological Disorders and Stroke, National Institutes of Health, Bethesda, MD 20892 USA; 2 University of Vienna, Vienna Austria; 3 Yale University, New Haven, CT USA; 4 School of Life Sciences, Guangzhou University, Guangzhou, 510006, China; 5 Theoretical Molecular Biophysics Section, National Heart, Lung and Blood Institute, National Institutes of Health, Bethesda, MD 20892 USA

## Abstract

The human serotonin transporter SERT facilitates serotonin (5-HT^+^) transport into cells by coupling to Na^+^ co-transport and K^+^ exchange. Although extracellular Cl^−^ is also essential for transport, whether Cl^−^ ions are transported has been disputed, raising the question why Cl^−^ ions are required? Here, we examine the role of Cl^−^ using transport measurements, conformational assays, and molecular simulations. We show that Cl^−^ is not transported and does not affect Na^+^-mediated cytoplasmic pathway closure but does reduce the accessibility of residues in the extracellular pathway, mimicking transport-related occlusion. Simulations indicate that Cl^−^ ion binding constrains the helices in the so-called bundle, but not interactions spanning the extracellular pathway thought to act as a molecular gate. We surmise that Cl^−^ (i) increases the stability of surrounding helices, (ii) enhances Na^+^ binding affinity, and (iii) decreases extracellular pathway accessibility, thereby facilitating transport-related conformational changes. These findings explain SERT’s requirement for chloride and highlight distinct features of proteins in the same neurotransmitter transporter family.

## Teaser

Decades-long question solved: Cl^−^ ions boost serotonin reuptake by limiting transporter dynamics, not by crossing the membrane.

## Introduction

Transporters can couple the energy provided by a transmembrane ion concentration difference to the energetically unfavorable accumulation of the transported substrate. This ability depends on the binding of required ligands to control the conformational changes that physically move the substrate and co-transported ions across the membrane. Serotonin transporter (SERT, SLC6A4), a member of the NSS (Neurotransmitter:Sodium:Symporter) family, catalyzes Na^+^ and Cl^−^-dependent accumulation of serotonin (5-hydroxytryptamine, 5-HT^+^) within serotonergic neurons, platelets and other cells, driven by the movement of Na^+^ into the cell and K^+^ out of the cell [1]. Other members of the NSS family include transporters for the neurotransmitters dopamine, norepinephrine, glycine, and GABA (γ-aminobutyric acid).

**Figure 1.**
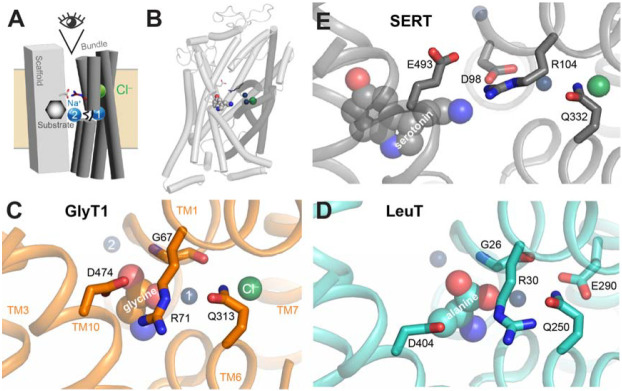
SLC6 transporter structures. **(A, B)** Schematic and cartoon representation of the LeuT fold, illustrating the central locations of the substrate, Na^+^ (*blue spheres*) and Cl^−^ ions (*green spheres*), and the position of the extracellular gate salt bridge residues (*sticks*). In the schematic (A), the bundle region, comprising transmembrane helices 1, 2, 6, and 7 is in dark gray, while the scaffold region is in light gray. **(C-E)** The extracellular interaction network viewed from the orientation indicated in panel A. The Gln-Arg-Asp/Glu network connecting the Cl^−^ binding site to helices in the scaffold is highlighted in sticks. In the amino acid transporters GlyT1 (C; PDB entry 8WFI) and LeuT (D; PDB entry 3F48), the key acidic residue in TM10 is an aspartate (Asp474 and Asp404, respectively), whereas in the monoamine transporter SERT (E; PDB entry 7LIA), Glu493 plays this role. In LeuT, the Cl^−^ ion is replaced by the side chain of Glu290. Protein is shown as cartoon helices viewed from the extracellular side. Ions and substrates are shown as spheres, and key residues are shown in stick representation and labelled using the numbering of the corresponding transporter. Note that the sequence numbering in GlyT1b here is that in the structure [21], UniProt identifier P48067-3, which is a different isoform from that referred to in the main text [20]. Amino acid substrates provide a carboxylate group to interact with a glycine in TM1 and the Na^+^ ion at the Na1 site, whereas the monoamine substrate interacts with an aspartate side chain in TM1 (Asp98). Structures are rendered using PyMol (Schrödinger, Inc).

Na^+^ stabilizes SERT in an outward-open conformation [2, 3] and the subsequent binding of Cl^−^ and 5-HT^+^ from the cell exterior transforms SERT into an inward-open conformation [4, 5] from which 5-HT^+^ and Na^+^ dissociate, thereby coupling their transmembrane movement [6]. To return to the outward-open state, cytoplasmic K^+^ binds [7-9], replacing Na^+^ and allowing outward-open reorientation of SERT. These conformational changes involve movement of helices in the so-called bundle (TM helices 1, 2, 6, and 7; Fig. 1A, B) as well as TM5 in the scaffold region, as indicated by the measured accessibility of cysteine residues placed into the cytoplasmic and extracellular pathways at positions accessible to aqueous reagents in one conformation and less accessible in another [10]. Although early results suggested that SERT also coupled Cl^−^ and 5-HT^+^ influx (symport) [11], more recent data indicates that intracellular Cl^−^ does not affect transmembrane currents associated with 5-HT^+^ transport, suggesting that intracellular release of chloride may not be coupled to that of 5-HT^+^ and Na^+^ [12]. Consequently, SERT might diverge from the NSS amino acid transporters for glycine (GlyT1) and GABA (GAT1), which couple substrate and Cl^−^ influx [13, 14]. Nevertheless, Cl^−^ is required for SERT function [15], raising the question of how Cl^−^ mediates its influence on SERT. One likely contribution is that Cl^−^ binding increases the affinity of SERT for Na^+^ [16]. Previous studies of GlyT1 revealed another influence of Cl^−^, namely on the conformational state of the transporter [17]. Specifically, in the presence of Na^+^, addition of either the substrate (glycine) or Cl^−^ alone led to a decrease in accessibility of the extracellular permeation pathway. However, these combinations of substrates resulted in much smaller changes in the cytoplasmic pathway, which is stabilized in a closed conformation by Na^+^. When both glycine and Cl^−^ were added together with Na^+^, there was a further decrease in accessibility of the extracellular pathway and a large increase in the cytoplasmic pathway, consistent with the conversion from outward- to inward-facing conformation. These results also suggested that substrate and Cl^−^ act independently to affect conformational change in GlyT1.

The accessibility data for GlyT1 suggest that Cl^−^ may influence the conformational state also for SERT. However, despite the similarities in sequence and structure between SERT and GlyT1, there are important mechanistic differences. For example, GlyT1 transports two Na^+^ ions and a Cl^−^ ion together in the step that delivers glycine to the cytoplasm, whereas SERT transports only one Na^+^ with 5-HT^+^ [6, 13]. Moreover, SERT utilizes the outwardly directed K^+^ gradient to drive the return step while GlyT1 does not depend on K^+^ [7, 18]. Substrate interactions also differ markedly. For amino acid transporters in the NSS family, such as GlyT1, an important interaction (first discovered in a bacterial amino acid transporter, LeuT) is formed between the substrate carboxyl group and one of the two bound Na^+^ ions (Fig. 1C, 1D) [19-21]. However, amine substrates of transporters like SERT, such as 5-HT^+^, lack this carboxyl and in NSS amine transporters like SERT, a protein aspartate side chain γ-carboxyl (as in Asp98 in SERT) occupies a similar position to that of amino acid substrates (Fig. 1E) [20, 22]. Other NSS amine transporters (for norepinephrine and dopamine; NET and DAT, respectively) also contain aspartate at the position corresponding to Asp98 in SERT [23-26], whereas all NSS amino acid transporters contain glycine at that position (Fig. 1C).

Given the known mechanistic differences between amino acid and monoamine transporters, we considered whether Cl^−^ acts similarly in GlyT1 and SERT. From a structural perspective, the interactions in the Cl^−^ binding site in NSS transporters are well conserved, as is the interaction network connecting it to the extracellular pathway (Fig. 1C-E). For example, a conserved glutamine residue (Gln332 in SERT) interacts with the bound Cl^−^ ion in both transporters [17, 27]. In LeuT (and GlyT1), this glutamine is close to a conserved arginine (Arg104 in SERT), which alternatively can salt bridge with an acidic residue across the extracellular pathway (Glu493 in SERT) [19, 22], depending on the orientation of the arginine side chain (Fig. 1C-E). Formation of this salt bridge is thought to contribute to closing the cytoplasmic pathway [28]. Thus, we proposed for GlyT1 that the effect of Cl^−^ binding shifted the arginine rotamer distribution toward formation of this salt bridge by favoring interaction of the arginine with the aspartate, rather than the glutamine (see Fig. 1C). Mutation of this glutamine to glutamate stabilized GlyT1 in an outward-open state (as measured by decreased accessibility of the cytoplasmic pathway) and was consistent with a stronger interaction between the arginine and glutamate, relative to the wild-type glutamine that it replaced. (Note that due to differences in sequencing and isoforms, Gln313 in Figure 1C corresponds to Gln299 in the work of Zhang et al. [17].)

Here, using similar accessibility measurements and molecular dynamics simulations, we examined whether Cl^−^ influences conformation in SERT in the same way as in GlyT1. The findings explain why Cl^−^ is required for transport and demonstrate which features unique to the sequence of SERT are important in mediating chloride’s effect.

## Results

### Coupling of transmembrane Cl^−^ concentration gradients to SERT-mediated 5-HT^+^ influx

**Figure 2.**
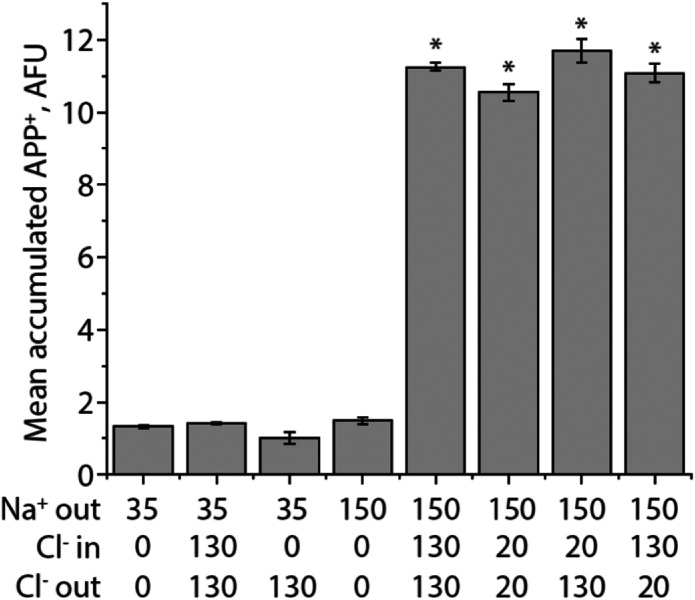
Influence of Na^+^ and Cl^−^ gradients on APP^+^ accumulation by reconstituted SERT proteoliposomes. The indicated concentrations of internal and external Na^+^ and Cl^−^ used were present during reconstitution of SERT proteoliposomes (*in*) which were diluted into assay medium (*out*), while maintaining an equal pH (7.4) on both sides of the membrane. Internal Na^+^ was held constant at 35 mM and all solutions were adjusted to pH 7.4 with 20 mM phosphate with NMDG^+^ and gluconate added to maintain the same ionic molarity while generating inward-directed gradients of Na^+^ and Cl^−^, as indicated. Asterisks indicate significantly different levels of APP^+^ accumulation, relative to those measured in the absence of Na^+^ gradient or at 0 Cl^−^ (columns 1-4) (*P < 0.05) using Student’s paired t test. All error bars represent the SEM; n = 3. None of the APP^+^ levels in columns 1-4 were significantly different from each other (P > 0.35). Differences between column 5 *vs.* 6, 7 or 8 were not significant (P > 0.44), and P values for the differences between columns 6 *vs.* 7, 6 *vs.* 8 and 7 *vs.* 8 were between 0.076 and 0.125.

Previous data regarding the ability of Cl^−^ gradients to act as a driving force for 5-HT^+^ accumulation by SERT were inconclusive. Experiments using platelet plasma vesicles suggested that a Cl^−^ gradient (out>in) could drive 5-HT^+^ accumulation based on the requirement for external, but not internal Cl^−^, and the expectation that 5-HT^+^ transport by SERT was electrically neutral [7], consistent with a symport mechanism in which 5-HT^+^ was transported stoichiometrically with one Cl^−^ ion [11] and one Na^+^ ion [6] followed by antiport of one K^+^ ion [7]. Subsequent electrophysiological studies have cast doubt on 5-HT^+^-Cl^−^ symport [12] prompting re-evaluation of this stoichiometry. We directly tested the ability of a Cl^−^ concentration gradient to drive substrate accumulation using SERT reconstituted into proteoliposomes [9]. The results in Fig. 2 show that, in the absence of a transmembrane Na^+^ gradient (leftmost 3 columns), no significant accumulation of the SERT substrate APP^+^ occurred when Cl^−^ was absent (column 1), present on both sides at equal concentrations (column 2), or present only outside the vesicles (column 3). The presence of a Na^+^ gradient (columns 4-8) alone was not sufficient to increase accumulation in the absence of Cl^−^ (column 4), but when Cl^−^ was present outside the vesicle, accumulation increased. This increase was similar in the presence of either 20 or 130 mM external Cl^−^ but was independent of the presence (compare columns 5 or 6 with 7 or 8) or direction (columns 7 vs 8) of a transmembrane Cl^−^ gradient. These results are inconsistent with SERT catalyzed Cl^−^-substrate symport.

### Conformational effects of Cl^−^ and 5-HT^+^

**Figure 3.**
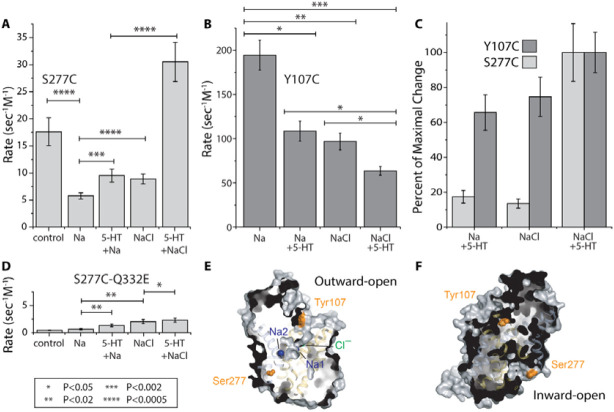
Accessibility changes in SERT induced by ions and substrate serotonin. (**A, B**) The conformational response to ligand binding was estimated by changes in the reactivity of Y107C in the extracellular pathway and S277C in the cytoplasmic pathway toward (2-(Trimethylammonium)ethyl)methanethiosulphonate, MTSET added to the medium of intact cells expressing SERT Y107C-C62A or to membrane samples from cells expressing SERT S277C-C62A. (**C**) Conformational response, in the presence of Na^+^, to Cl^−^ or 5-HT^+^, shown as relative to the response to both. The change in accessibility of Y107C and S277C between Na^+^ alone and NaCl with 5-HT^+^ was set to 100% and the changes induced by Cl^−^ or 5-HT^+^ scaled accordingly. Light grey bars represent cytoplasmic pathway (S277C) reactivity and dark grey bars, that of the extracellular pathway (Y107C). All error bars represent the SEM; n ≥ 4. **(D)** S277C reactivity with additional mutant Q332E. Asterisks indicate significantly different rate constants (*P < 0.02; **P < 0.01) using Student’s t-test. Error bars represent the SEM; n ≥ 4. (**E, F**) Surface exposure of residue Tyr107 and Ser277 (orange spheres) in human SERT depends on the conformational state, as shown for outward-facing (E) and inward-facing (F) conformations, PDB entries 7LIA and 6DZZ, respectively, viewed from within the membrane plane. The protein is shown as surface and helices, with the bundle helices (TM1, 2, 6, 7) in yellow, and other helices in gray; Na^+^ (*blue*) and Cl^−^ ions (*green*) are shown as spheres where present.

To assess conformational changes in SERT, we measured the reactivity of cysteine residues replacing either Ser277 in the cytoplasmic pathway or Tyr107 in the extracellular pathway (Fig. 3), corresponding to residues we have shown to be sensitive to conformational changes in LeuT, GlyT1, and SERT [10, 17, 20]. Specifically, Tyr107 was more accessible to aqueous reagents in outward-facing conformations (Fig. 3E) and Ser277 was more accessible in inward-facing conformations (Fig. 3F). SERT-S277C reactivity was decreased in the presence of Na^+^, as previously reported [2], due to the ability of Na^+^ to stabilize the cytoplasmic pathway in a closed conformation in SERT and other NSS transporters [3, 10, 17] as shown in Fig. 3A (column 1-2). In the presence of Na^+^, addition of either 5-HT^+^ (column 3) or Cl^−^ (column 4) partially reversed the effect of Na^+^, and addition of both 5-HT^+^ and Cl^−^ further increased cytoplasmic pathway accessibility to an extent greater than in the absence of Na^+^ (column 5).

In the extracellular pathway, the response to 5-HT^+^ and Cl^−^ was opposite to that of the cytoplasmic pathway (Fig. 3B). In the presence of Na^+^ (column 1), addition of 5-HT^+^ markedly decreased accessibility of a cysteine replacing Tyr107 (column 2) and addition of Cl^−^ had a similar effect (column 3). Addition of both 5-HT^+^ and Cl^−^ further decreased accessibility (column 4). Although the maximum accessibility changes in either the extracellular and cytoplasmic pathways occurred in the presence of both 5HT^+^ and Cl^−^, the fraction of that maximum change resulting from addition of either 5-HT^+^ or Cl^−^ was quite different between the pathways. Fig. 3C shows the change in accessibility relative to the maximum. Addition of 5-HT^+^ or Cl^−^ changed cytoplasmic accessibility (light grey bars in the first two pairs of columns) by only about 15-20% of the maximum observed with both ligands, but they changed accessibility of the extracellular cysteine by 65-70% of the change induced by both ligands together (dark gray bars in the first two pairs of columns).

### Behavior of an extracellular pathway network depends on the state of the transporter

To provide a molecular-level understanding of these findings, we turned to MD simulations. In our previous study using LeuT [17], we found that two conserved residues, Arg30 and Gln250 (corresponding to Arg104 and Gln332 in SERT, respectively, Fig. 1) interacted consistently in the outward-open state, but not in the outward-occluded state, where Arg30 instead salt-bridges to Asp404, forming the so-called extracellular gate (Fig. 1D). Thus, this salt bridge appears to be a feature or influencer of the degree of extracellular closure (and consequently also of intracellular opening). Note that in those simulations, the nearby Glu290 in LeuT was charged to mimic a Cl^−^-bound state. Based on these observations, it was proposed that Cl^−^ mediates salt-bridge formation and pathway closure, and does so *via* the conserved Gln residue [3, 17, 29].

**Figure 4.**
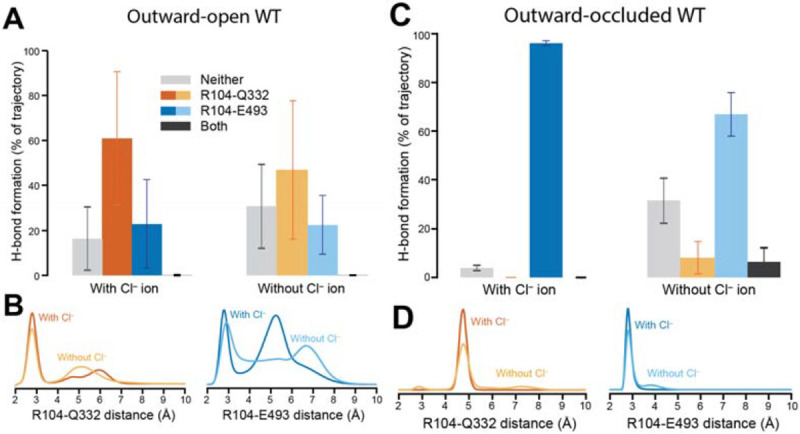
Behavior of the interaction network connecting the Cl^−^ binding site to the extracellular pathway salt bridge in wild-type SERT. Molecular dynamics simulations were initiated with **(A, B)** outward-open and **(C, D)** outward-occluded conformations for 0.5 μs (n=4 or n=8, respectively). Substrate 5-HT^+^ and two Na^+^ ions were placed within their respective sites. **(A, C)** Formation of a hydrogen bond (distance <3.2 Å) between any donor atom of Arg104 and an acceptor atom of either Gln332 (orange bars), Glu493 (blue bars) or both (black bars), either in the presence (left) or absence (right) of a Cl^−^ ion initially placed at its reported binding site. Gray bars represent the fraction of time that neither H-bonds are formed. Error bars reflect standard deviations across repeat trajectories. In (**B, D**) the distance was measured either in the presence (dark lines) or the absence (pale lines) of a Cl^−^ ion.

**Figure 5.**
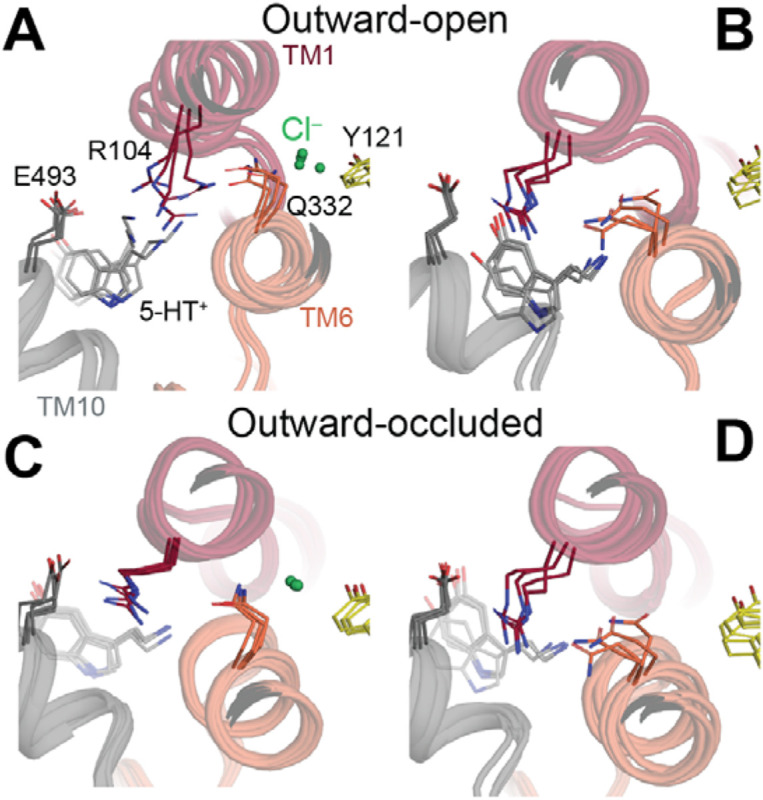
Interaction network connecting the extracellular pathway with the Cl^−^ site in SERT. Simulation frames are viewed from the extracellular side, as indicated in Figure 1A. Multiple frames were taken from simulations of the **(A, B)** outward-open and **(C, D)** outward-occluded conformations, either in the presence (A, C) or absence (B, D) of a Cl^−^ ion (*green sphere*), with TM1 (*red*), TM6 (*orange*) and TM10 (*gray*) shown in cartoon representation. Key residues and the substrate 5-HT^+^ (*grey*) are shown as sticks. Y121 from TM2 is colored yellow.

Consistent with those findings in LeuT, in simulations of SERT with bound 5-HT^+^, Na^+^, and Cl^−^, Arg104 interacted with Gln332 in the outward-open state (Fig. 4A-B; Fig. 5A) but not in the outward-occluded state (Fig. 4C-D; Fig. 5C). Further consistency with LeuT is seen in that Arg104 formed a salt bridge with the equivalent acidic residue, Glu493, more frequently in the outward-occluded state (Fig. 4C-D; Fig. 5C) than in the outward-open state (Fig. 4A-B; Fig. 5A). These simulations of Cl^−^-bound SERT in different conformations therefore support the idea that Gln332 senses the binding of Cl^−^ ions in SERT and thereby influences closure of the extracellular pathway salt-bridge.

### Behavior of the extracellular pathway in the absence of a Cl^−^ ion

In LeuT, the absence of chloride was, in one study, mimicked by protonation of Glu290 [29], which is an imperfect analog. Here, we aimed for a more direct comparison by analyzing simulations of SERT either with or without the bound Cl^−^ ion, in different states.

**Figure 6.**
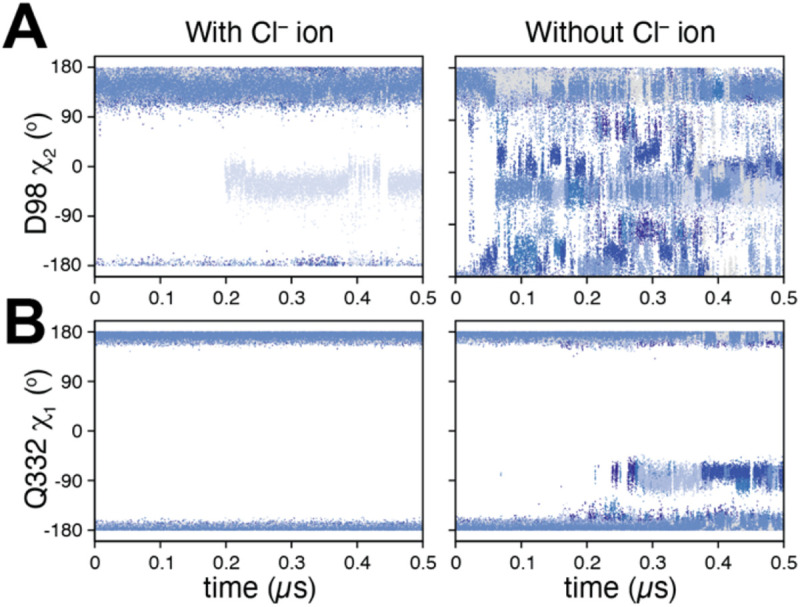
Dynamics of ion binding site side chains in the outward-occluded conformation of SERT depend on the presence of the Cl^−^ ion. **(A)** Asp98 and **(B)** Gln332 dynamics, measured as the torsion angle of the side chain χ_1_ or χ_2_ dihedrals, respectively, shown as a function of simulation time for n = 8 trajectories in the presence (*left column*) and absence (*right column*) of a Cl^−^ ion in its binding site. Each trajectory is colored a different shade of blue.

In simulations of SERT with the extracellular pathway open, the network behaved similarly whether Cl^−^ was bound or not (Fig. 4A-B), with the Arg104-Glu493 salt bridge formed around 20% of the simulation time. Thus, the anion did not substantially alter the local network in the outward-open state. By contrast, in simulations with the extracellular pathway closed (Fig. 4C-D), the local network was influenced by the ion: specifically, Gln332 was more dynamic in the absence of the Cl^−^ ion (Fig. 5C-D), accessing gauche(+) χ_1_ side chain angle conformations (Fig. 6B) in which it interacted with Arg104 (Fig. 5D). Remarkably, however, Arg104 was able to salt bridge with Glu493 even when a Cl^−^ ion was absent (Fig. 4D; Fig. 6**C-D**, *blues*). These observations suggest that despite stabilizing the local network more in the occluded state than in the outward-open state (and therefore favoring occlusion), the anion does not act exclusively via Gln332-mediated enhancement of salt-bridge formation in SERT, as previously proposed.

### Chloride acts on features other than the extracellular salt bridge in SERT

To identify potential broader consequences of the absence of a Cl^−^ ion that could further destabilize the occluded state and lead to the measured accessibility changes, we characterized the dynamics of nearby residues. The most notable dynamics were observed for Asp98 (highlighted in Fig. 1E), whose side chain adopted a wide range of conformations in the Cl^−^-free transporter (Fig. 6A), despite being within salt bridge distance of the charged primary amine of the bound 5-HT^+^ ion and, on the other side, to the Na1 ion.

**Figure 7.**
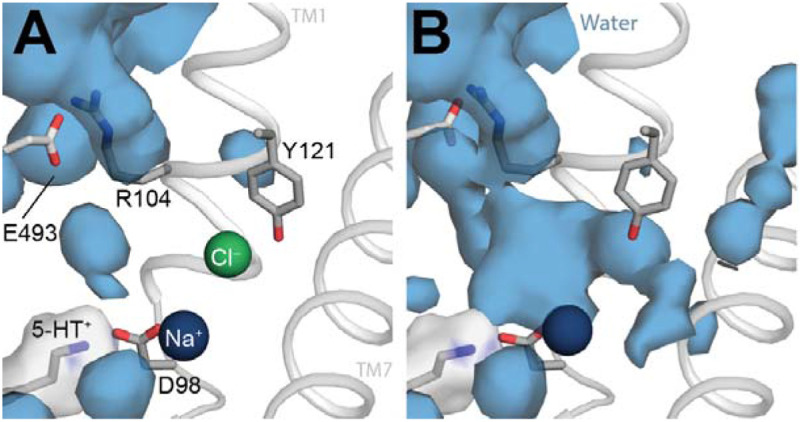
Hydration of the outward-occluded conformation of SERT increases in the absence of a Cl^−^ ion. Water occupancy maps (*blue surface*) are shown with an equilibrated conformation of SERT as reference, for Molecular Dynamics simulations of the outward-occluded state in **(A)** the presence and **(B)** the absence of a Cl^−^ ion (*green sphere*). Occupancies within 20 Å of Asp98 were averaged over n = 8 trajectories, respectively. Selected side chains and 5-HT^+^ are shown as sticks, along with the Na^+^ ion in the Na1 site (*blue sphere*) and the occupancy of 5-HT^+^ (*white surface*).

The increased dynamics relative to the Cl^−^-bound protein coincide with greater hydration of this region (Fig. 7). Specifically, water molecules from the extracellular side solvate not only the empty Cl^−^ site, but also the extracellular salt bridge region of the pathway, and other regions within the bundle helices (Fig. 7B). We therefore posit that the increased conformational freedom exemplified by Figure 5 reduces the probability of pathway closure when Cl^−^ ions are not available to bind. These observations align with the cysteine labelling data (Fig. 3): for wild-type SERT in the presence of Na^+^ alone, or both Na^+^ and 5-HT^+^, the addition of Cl^−^ reduced the accessibility of Y107C in the extracellular pathway. These effects are likely to be additive with the reduced Na^+^ binding affinity measured in low Cl^−^ concentrations implied by the data in Tavoulari et al [16]. Overall, the combination of three distinct effects: (i) reduced sodium affinity; (ii) reduced salt-bridge formation, and (iii) reduced stability of nearby sidechains, significantly diminishes the probability of forming the occluded conformation in the absence of chloride, likely increasing the kinetic barrier to transport.

### Conformational consequences of mutating Gln332 to glutamate

In previous work with GlyT1, the role of chloride-mediated salt bridge formation was tested by mutation of Gln299 (corresponding to SERT Gln332, Fig. 1) to glutamate. Specifically, it was proposed that a negatively charged carboxyl side chain at this position would sequester Arg57 (Arg104 in SERT) and prevent it from establishing the salt bridge with Asp460 (Glu493 in SERT). Indeed, in GlyT1, this mutation led to a dramatic decrease in the accessibility of the cytoplasmic pathway, as if the transporter were strongly biased toward inward-closed conformations. We tested the corresponding mutation in SERT, with similar results (Fig. 3D) (note the similar y-axis scales in Figs. 3A and 3D). As in GlyT1, this mutation and the resulting conformational effects ablated transport activity, while ligand affinity was preserved, allowing for measurements of cytoplasmic pathway accessibility. Although the overall accessibility of the cytoplasmic pathway was markedly reduced under all conditions – and the Na^+^-dependent decrease seen in Fig. 3A was not observed (Fig. 3D, columns 1 vs 2) – small but significant increases in accessibility were found in the presence of Na^+^ when either 5-HT^+^ or Cl^−^ was added (Fig. 3D, columns 2 vs. 3 and 4) with a less significant increase with both 5-HT^+^ and Cl^−^ (Fig. 3D, column 5 vs. 4).

### Impact of the Q332E mutation on the molecular network involving Arg104

The cysteine reactivity of Q332E indicates that the extracellular pathway is more likely to be open than in wild-type SERT, or rather that the intracellular pathway is more likely to be closed; yet, puzzlingly, the mutant retained sensitivity to Cl^−^ (Fig. 3D). To analyze how a glutamate side chain at position 332 affects the molecular network, we repeated the simulations with Gln332 mutated to glutamate in the presence of 5-HT^+^, Na^+^, and Cl^−^ ions. As envisioned, charged Glu332 interacted more frequently than Gln332 with Arg104; however, this was true only when the pathway was outward-open (Fig. S3A left panel; Fig. S3B, dark lines) and not for the occluded state (Fig. S3C, left panel, Fig. S3D, dark lines. Moreover, the Cl^−^ ion placed next to charged Glu332 was inherently unstable and typically left the site within the 500 ns simulation time frame (Fig. S4B; green lines, n=4). This contrasts starkly with the stable coordination observed in wild-type SERT (Fig. S4A; green lines, n=8). We attribute the rapid unbinding of the Cl^−^ ion in Q332E to electrostatic repulsion between the Cl^−^ ion and the charge at Glu332, which will be substantial even when Glu332 is rotated away and interacting with Arg104.

We also note that the presence of a Cl^−^ ion profoundly affects the Na^+^ ion at the neighboring Na1 site (Fig. S4A, orange lines; Fig. S4B, blue lines), which unbinds rapidly once the Cl^−^ site is empty, in both wild-type and Q332E SERT.

Given the instability of the bound Cl^−^ ion, to study the role of Glu332 in the local network, we repeated the simulations without the Cl^−^ ion. If Gln332 was indeed the primary mediator of Cl^−^ binding, we expected that the pathway salt bridge would be less frequently formed in the Glu332 mutant in the absence of Cl^−^ than in wild type. Indeed, when the pathway was open, the Arg104-Glu493 salt bridge was less favored (Fig. S3A, S3B, blue bars and lines) than for wild-type SERT (Fig. 4A, 4B, blue bars and lines). However, in the occluded state we did not observe a substantial reduction in pathway salt-bridge formation relative to the wild-type protein (blue bars in Fig. S3C *cf.* Fig. 4C). Thus, the mutation primarily influences the more outward-open states of the transporter in the absence of Cl^−^, stabilizing those conformations and presumably, therefore, inhibiting closure of the extracellular pathway, in line with the lack of measured cytoplasmic accessibility of S277C-Q332E (Fig. 3B).

In summary, our simulations suggest, first, that Glu332 reduces the affinity for Cl^−^ in the outward-open state relative to Gln332 (Fig. S4), and secondly that in the absence of the ion, the acidic carboxyl side chain in Glu332 strongly attracts Arg104 away from Glu493 (Fig. S3A, right panel), which in turn increases the energetic barrier to closure of the extracellular pathway (Fig. S3B, light blue). In wild-type SERT, by contrast, the Cl^−^ ion appears to act primarily through stabilization of a local network of interactions around Na1 required for formation of the occluded state.

## Discussion

Several observations reported here provide deeper understanding of the unique properties and mechanism of ion-coupled transport of 5-HT^+^ and possibly also of dopamine and norepinephrine. In contrast to two other well-characterized NSS family members, GAT1 and GlyT1, which transport amino acid neurotransmitters, we show that SERT does not symport its substrate with Cl^−^.

Another distinct feature of SERT is that it symports 5-HT^+^ with only 1 Na^+^ ion, despite having 2 Na^+^ binding sites, while GAT1 and GlyT1 symport 2 Na^+^ ions with substrate. The single transported Na^+^ ion almost certainly dissociates from the Na2 site, as that is the site to which intracellular K^+^ binds prior to the return of SERT to an outward-facing conformation [9]. The special property of monoamine transporters that prevents Na^+^ bound at the Na1 site from dissociating with substrate is apparently that these transporters, as the only NSS members that transport amine substrates, contain an Asp residue (Asp98 in SERT) that coordinates Na^+^ at Na1 through its side-chain carboxyl group, whereas NSS amino acid transporters, including GAT1 and GlyT1 have a glycine at the position corresponding to SERT Asp98, and use the substrate carboxyl group to coordinate Na^+^ at Na1. Substrate dissociation from GAT1 or GlyT1 removes this coordination and likely facilitates Na^+^ dissociation from the Na1 site. In turn, the loss of the cation from the Na1 site would be expected to destabilize an adjacent Cl^−^ ion (Fig. S4), providing an explanation for why GAT1 and GlyT1 also symport chloride.

Nevertheless, Cl^−^ is required for SERT function even though it isn’t transported. This requirement for Cl^−^ is similar to the requirement for an acidic amino acid in the bacterial NSS transporters LeuT and TnaT, which contain a Glu or Asp, respectively, where Cl^−^-dependent NSS transporters contain a serine residue that coordinates Cl^−^ [16, 27, 30].

Building on a previous study that highlighted the co-dependence of Na^+^ and Cl^−^ affinities [16], our simulations show a strong synergy of binding between the Na1-site Na^+^ ion and the bound Cl^−^ providing an explanation for the absence of intracellular Cl^−^ dissociation together with 5-HT^+^. We also show that binding a Cl^−^ ion influences the conformational dynamics of the extracellular side of the transporter. Surprisingly however, the salt-bridge spanning the extracellular pathway remains formed in all simulated states, even in the outward-open state, despite contrary observations for LeuT [20]. Thus, in SERT, Cl^−^ does not induce salt-bridge formation mediated via Gln332 as found for LeuT [16, 29] and suggested for GlyT1 [20].

To understand why the salt-bridge in SERT can remain formed in states where the extracellular pathway is open, it is important to note that the carboxyl is, uniquely, provided by a glutamate side chain, whose longer side chain apparently allows it to reach further across the pathway than the aspartate observed in most other NSS monoamine transporters (see Promals3D sequence alignment [31] in Fig. S5). Thus, even when the bundle helices, including TM1, are angled away from the scaffold, which includes TM10, the interaction remains formed.

We note that SERT (also uniquely) contains a second glutamate at the neighboring position in TM10 (Glu494; Fig. S5), which creates a more electronegative environment in the extracellular pathway. Analysis of the distances over time revealed that Arg104 can reach salt-bridging distance to Glu494, but only while also contacting Glu493 (data not shown). Thus, extracellular pathway closure is clearly more complex in SERT than in other NSS transporters where bundle-scaffold interactions appear more dependent on the TM1-TM10 salt-bridge interaction.

**Figure 8.**
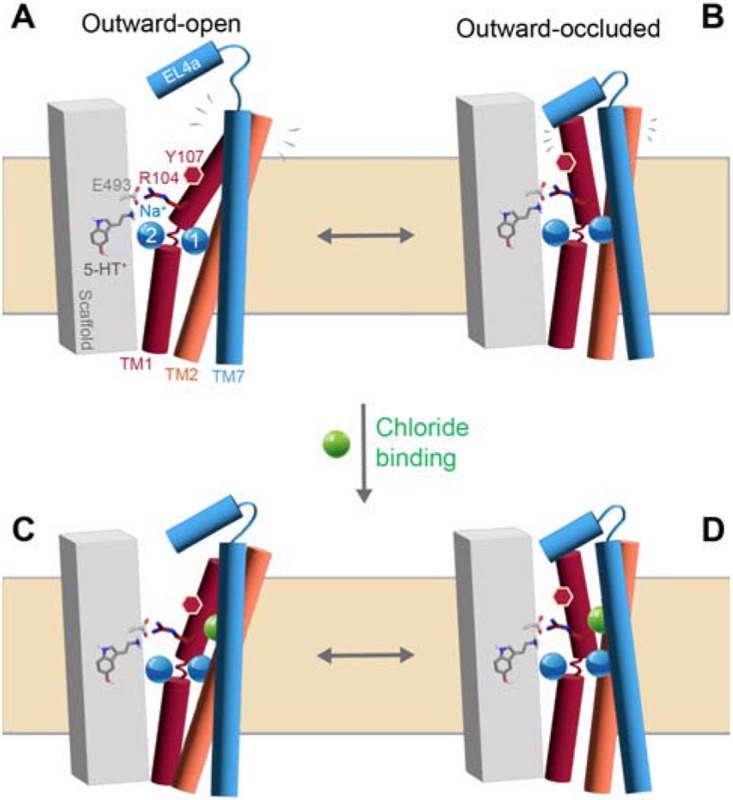
The chloride ion organizes and stabilizes the packing in the four-helix bundle. Schematics of the scaffold region (*gray box*) and the four-helix bundle (TMs 1, 2, 7 are shown as red, orange, and blue cylinders, respectively; TM6 is hidden for clarity) highlighting the differences in structural dynamics and the degree of Tyr107 exposure (red hexagon in TM1) in the presence **(C, D)** and absence **(A, B)** of chloride (*green sphere*). Serotonin is shown as gray sticks, the sodium ions bound (Na1 and Na2) as blue spheres.

The simulation analysis illustrates a significant reduction in helix packing when chloride is not bound, which allows water to enter between the helices (Fig. 6B). Given this, and the moderate rates of reactivity measured for Y107C in the presence of NaCl and 5-HT^+^ (Fig. 3A middle plot, right bar), we wondered how the solvent accessibility of the side chain at position 107 was changing in the different conformational states of the wild type protein simulated here. Indeed, even in the outward-occluded conformation with Cl^−^ bound, the mean and standard deviation of the surface area of Tyr107 accessible to an MTSET-like probe was 363 ± 8 Å^2^ (Table 1). Thus, despite the nearby extracellular loop 4 (EL4), the side chain retains significant accessibility, consistent with the measured rates (Fig. 3A middle plot, right bar). Nevertheless, Cl^−^ did influence the reactivity at Y107C (Fig. 3A); thus, we extended this analysis to the other states of the transporter (Table 1), which revealed a subtle increase in solvent accessibility for the Cl^−^-free occluded state, but a significant increase to 382 ± 32 Å^2^ and 410 ± 22 Å^2^ for the outward-open conformation in the presence and absence of the Cl^−^ ion, respectively. Thus, the trend follows that measured using MTSET: Tyr107 is more reactive in outward-facing conformations, but Cl^−^ reduces the reactivity of this region of the transporter. The induced change in the conformational dynamics, however, is more subtle than the major conformational change to the inward-facing state. We posit that the Cl^−^ ion acts to organize and stabilize the packing in the four-helix bundle comprising TM helices 1, 2, 6, and 7 even in outward-facing states of the transporter (Fig. 8).

When examining the mutant Q332E, we observed that Cl^−^ rapidly unbinds from the outward-open state, suggesting a very weak affinity. How then can we explain that NaCl modulates the reactivity of S277C experimentally in the Q332E mutant (Fig. 3B)? We posit that a small population of protonated Glu332 (due to a shift in pK_A_ at high Cl^−^ concentrations) will allow a low level of Cl^−^ binding. To test this proposal computationally would require simulations with Glu332 allowed to protonate dynamically during the trajectory, which is beyond the scope of the current study. A classical MD simulation of a protonated Q332E variant was also considered, but set aside for now, due to the introduction of further uncertainty.

To conclude, a combination of accessibility measurements and simulations answers a decades-long question regarding the behavior of SERT, with unexpected complexity and characteristics distinct from other neurotransmitter transporters. The unique behaviors of SERT may originate from slight differences in sequence in the extracellular pathway, highlighting the care that must be taken in inferring specific behaviors based on those observed in homologous transporters.

## Materials and Methods

### Cysteine Accessibility Measurements.

Conformational changes were measured using the accessibility of cysteine residues placed in the cytoplasmic (S277C) and extracellular (Y107C) permeation pathways as described previously [10]. For measurement of extracellular pathway accessibility, measurements were made with intact cells expressing rat SERT Y107C in the SERT C109A background [32] growing in 96-well culture plates. For cytoplasmic pathway accessibility, measurements were made with membranes prepared from cells expressing SERT S277C in the X5C background (a SERT mutant in which the five most reactive cysteine were mutated, C15A/C21A/C109A/C357I/C622A) on filters in 96-well filtration plates. A radioligand (β-CIT) binding assay [33] was used with membranes from disrupted cells because MTSET does not penetrate through intact cell membranes [34] and consequently does not access the cytoplasmic pathway in intact cells. In both cases, accessibility was measured by the rate of cysteine reactivity with MTSET, as described previously [10]. 5-HT^+^, where added, was present at 10 μM. NMDG^+^ was used to replace Na^+^ and gluconate replaced Cl^−^. The MTSET concentration causing half-maximal inactivation was determined and used to calculate the rate constant for cysteine modification, as described previously [4, 35]. MTSET concentrations were calibrated using Ellman’s reagent (5,5′-dithiobis(2-nitrobenzoate)) [36].

### Reconstitution of SERT Proteoliposomes.

Detergent-free reconstitution of SERT proteoliposomes was performed as described previously [9]. In brief, confluent HEK293T cells stably expressing human SERT wild-type (UniprotKB identifier: P31645) with a 10-His epitope at its C terminus were collected and disrupted by sonication and the cell membrane fraction solubilized in diisobutylene maleic acid (DIBMA) buffer and the native nanodiscs containing SERT were purified on Ni-NTA agarose and Sephadex G-200 size exclusion chromatography to remove imidazole. Lipids were sonicated and diluted into 20× volumes of the indicated internal buffer, followed by freeze-thaw and extrusion to produce unilamellar vesicles equilibrated with the internal buffer. Purified SERT in native nanodiscs were then added for a protein/lipid ratio of 1:100 (w/w), vortexed for 1 min, and incubated for 20 min on ice.

### Transport Assay with SERT Proteoliposomes.

To measure transport by reconstituted SERT proteoliposomes, 4-(4-(dimethylamino)-phenyl)-1-methylpyridinium (APP^+^) uptake was initiated by diluting 20 μL of SERT proteoliposomes preloaded with an internal buffer into 380 μL of an external buffer containing 2 μM APP^+^. After incubation for 1 min at 22 °C, the reaction was quenched by a rapid addition of 500 μL of ice-cold external buffer. APP+ remaining in solution was removed by filtration and the accumulated APP^+^ was measured by the fluorescence of the liposomes on the filters. All liposome-based APP^+^ uptake experiments were performed at least three times. Nonspecific uptake was measured in the presence of 10 μM fluoxetine.

### Ab initio quantum chemical calculations.

Quantum chemical calculations were performed to generate reference data for the optimization of a CHARMM General Force Field (CGenFF) for 5-HT^+^ (**see** Supporting Information). Calculations were performed in the gas phase using Gaussian 16 [37]. Following CGenFF parametrization procedures [38, 39], geometry optimization of 5-HT^+^ and potential energy surfaces generated by relaxed scans of the dihedral angles of rotatable bonds in the ion, were performed with MP2/6-31G(d). The calculated potential energy surfaces were used as targets to adjust dihedral angle parameters. Atomic charges were refined by targeting the interaction energies and H-bond distances in various 5-HT^+^-H_2_O complexes. These complexes are generated by placing a single H_2_O molecule near the different H-bond donor (C-H, N-H, O-H) or acceptor (O) sites in 5-HT^+^. The complexes were set up with a linear H-bond angle between 5-HT^+^ in its MP2/6-31G(d) optimized geometry and a water molecule in its TIP3P geometry (*r*_O-H_ = 0.9572 Å, *θ*_HOH_ = 104.52°) [40]. The interaction distance in these complexes was then optimized at the HF/6-31G(d) level while keeping all other degrees of freedom fixed. The interaction energies of these complexes were calculated without correction for basis set superposition error and together with the H-bond distance were used as target data to adjust the atomic charges on the ring heavy atoms of 5-HT^+^.

### Molecular dynamics simulations.

Simulations of two conformations of human SERT were prepared according to a protocol described previously [41]. The structure of an outward-occluded conformation was based on a carefully curated hybrid model [42], while an outward-open conformation was based on a structure (Protein Database, PDB identifier 5I71 [43]) including the two reported Na^+^ ions in sites Na1 and Na2, plus a Cl^−^ ion assigned the position reported in another structure (PDB entry 5I6X) [43]. Note that this work was initiated before the release of serotonin-bound structures [22]. 5-HT^+^ was added to the S1 binding site according to the position obtained from Induced Fit docking with Schrödinger [42]. Force field parameters for 5-HT^+^ were developed as described below. Residue Glu508 was set to be protonated, based on proximity to Glu136 and the results of our Poisson-Boltzmann calculations to predict the protonated partner of the pair [44] and residues Cys200 and Cys209 were disulfide bridged. Hydrogen atoms in the protein and cavity-bound water were energy minimized for 250 steps using, first, steepest descents and then conjugate gradient protocols. In both cases, the structures were also converted to a coarse-grained representation according to the Martini v2.2 force field [45] and then inserted into a coarse-grained hydrated palmitoylolyeoylphosphatidylcholine (POPC) lipid bilayer at a salt concentration of 150 mM NaCl. Lipids and salt solution were equilibrated around the protein using Gromacs v2018.8 [46] for 50 μs, while constraining the secondary structure of the protein, as described in [47].

To determine an approximate box size, we analyzed the perturbations of the membrane, finding them to be minimally impacted by the periodic boundary images at a size of 12 x 12 x 11 nm. A representative snapshot with minimal structural deviation from the average equilibrated bilayer was then converted to an all-atom system [48]. The protein was replaced by the energy-minimized atomistic sodium- and chloride-bound model and cavities therein were solvated with Dowser [49]. Subsequent simulations were carried out with NAMD v2.14 using the CHARMM36m force field [50] and TIP3P waters [40].

The periodic box contained ~150,000 atoms with dimensions of ~11 nm^3^ after coarse-grained equilibration. Two additional wild-type all-atom systems were created by removal of the bound chloride ion which required removal of a bulk sodium ion to balance the net charge of the system.

Structures of Q332E-SERT were derived from the wild-type structure by replacement of the Gln332 side-chain amide group with an O atom. To preserve the net charge, a Na^+^ ion was added to the bulk solution.

Equilibration of the coarse-grained protein-lipid system was carried out in multiple stages according to [41]. In brief, after conversion to the all-atom representation, the lipids, waters, and ions were energy minimized for 100 steps using the conjugate gradient algorithm. To ensure that lipids would not be trapped within protein aromatic rings, pseudo-atoms were placed in the center of each ring during a second 100-step energy minimization. Subsequently, the system was energy minimized for 5,000 steps using the conjugate gradient algorithm with positional restraints on the protein backbone, the nonhydrogen atoms of the side chains, and the O atoms of cavity waters. During a final 500 steps of conjugate gradient minimization, we applied a protein center-of-mass restraint and dihedral restraint on the and ψ and χ_1_ angles, with a force constant of 256 kJ/mol. The ions present in the protein binding sites and the Arg104-Glu493 salt bridge were restrained to a minimum number of coordinating interactions using the COLVARS module [51, 52]. Specifically, atoms coordinating the ions (Ala96 O, Asn101 O_δ1_, Ser336 O, Ser336 O_γ_, Asn368 O_δ1_ in Na1, Gly94 O, Val97 O, Leu434 O, and Ser438 O_γ_ in Na2; and Asn101 N_δ2_, Tyr121 O_η_, Gln332 N_ε2_, Ser336 O_γ_, and Ser372 O_γ_ in the Cl^−^ site) were constrained to ≤2.5 Å from a Na^+^ ion or ≤3.5 Å from a Cl^−^ ion, with a force constant of 10 kcal/mol and a minimum coordination number of 2. The extracellular pathway salt bridge comprising Arg104 and Glu493 (between Arg104 N_ε_, N_η1_ or N_η2_ and Glu493 O_δ1_ or O_δ2_) was restrained to ≤3.5 Å, with a force constant of 50 kcal/mol and a minimum coordination number of 1. The 5-HT^+^ present in the S1 site was restrained with distances ≤2.8 Å between Asp98 O_δ2_ and the cationic nitrogen of 5-HT^+^, as well as Thr439 O_γ1_ and the hydroxyl group of 5-HT^+^, with a force constant of 200 kcal/mol. The same restraints were applied during six subsequent stages of molecular dynamics (MD) equilibration lasting >204 ns in total, while progressively reducing the force constants (1). The salt bridge restraint was kept at a force constant of 50 kcal/mol during the first 4 ns, and then progressively reduced by 10 kcal/mol per stage until reaching 10 kcal/mol at equilibration stage 6.

MD simulations were carried out using a 2 fs time step and trajectory output was saved every 4 ps for analyses. A temperature of 298 K was maintained using Langevin Dynamics, and pressure was kept at 1.01325 bar (oscillation time scale = 200 fs; damping scale = 50 fs) using Nosé–Hoover Langevin piston pressure control [53]. A cut-off distance of 12 Å with a switching function starting at 10 Å was used for Van der Waals interactions. The particle–mesh Ewald method [54] was used for long-range electrostatic forces. Multiple production runs, each ~0.5 μs long, were carried out without any restraints applied, but with different initial velocities.

### MD Simulation Analysis.

Distance and dihedral angle data were calculated with COLVARS module v2022-05-24 [52] in VMD v1.9.3 [55]. The calculated data were visualized and plotted using python matplotlib v3.9.3 [56]. Occupancy maps of ions and water were calculated in VMD using the Volmap plugin v1.1 and visualized in VMD and PyMOL version 3.1.3 (Schrödinger Ltd).

## Supplementary Material

Supplement 1

## Figures and Tables

**Table 1:** Surface accessibility of Tyr107 during MD simulations of SERT

Conformation	Is Cl^−^ bound?	Mean (Å^2^)	Stddev	Num of traj
Outward-open	−	410	22	4
	+	382	32	4
Outward-occluded	−	369	12	8
	+	363	8	8

Outward-facing state simulations are with 5-HT^+^ and Na^+^. The surface is computed by rolling a large probe approximating, MTSET, with radius 3 Å over the surface of all atoms at each time step and averaging over the timesteps in each trajectory.
