# Supplementary material for: The Role of Chloride Ions in Serotonin Transport
Source: bioRxiv. 2025 May 20:2025.05.20.654092. Preprint. [Version 1] doi: 10.1101/2025.05.20.654092 (PMC12139788; doi:10.1101/2025.05.20.654092)
Supplement: Supplement 1 [file media-1.pdf]

Supporting Information

Supplementary Methods

Development of force field parameters for serotonin

Generation of generalized initial parameters – The CGenFF program website (https://cgenff.silcsbio.com) was used to build a CGenFF model for 5-HT$^+$, compatible with the CHARMM force field (**Fig. S1A**). The program performs atom typing and then assigns parameters by analogy with existing groups, while assigning a penalty score to each parameter. For 5-HT$^+$, parameters with penalty scores > 10 were associated with certain ring heavy atoms and with the quaternary N atom. While penalties of the dihedral angle parameters were low (< 5), we tested their impact on the energy surface for dihedral rotation (**Fig. S1B-D**), compared to energies computed using MP2/6-31G(d) quantum chemical calculations (see Methods).

Ab initio geometry optimization with MP2/6-31G(d) revealed two stable structures for 5-HT$^+$ that differ in the $C_7C_{17}C_{20}N_{23}$ dihedral angle (**Fig. S1C, blue**), leading to a 7.3 kcal/mol difference in energy, as well as a significant difference in molecular dipole moment (**Table S1**). The default CGenFF model parameters predict correctly which conformer is more stable (**Fig. S1C, gray, Table S1**), and the orientation of the dipole (not shown), but the relative magnitude of the energies differs.

Optimization – The atomic charges of the $CH_2NH_3^+$ fragment were set to those in the ethylammonium ion in CGenFF and the charge on the other CH_2 group was set to that of typical CH_2 groups (q = –0.18e for C and 0.09e for each H). The atomic charges of other H atoms and of O were left unchanged from the default CGenFF values. We then tested whether the parameters reproduce the interactions of 5-HT$^+$ with water. Specifically, twelve 5-HT$^+$-H_2O geometries were optimized using MP2/6-31G(d) (**Fig. S2**); the H-bond distances and interaction energies of these complexes are reported in **Table S2**. The average unsigned error in the default CGenFF parameters relative to the QM values is 12% and 7% for the interaction energies and the H-bond distance, respectively. Charges on ring heavy atoms were adjusted to better reproduce the interaction energies. As a consequence, the average unsigned error of the interaction energies reduced to 6%, while the H-bond distance error remained at 7%. Moreover, the potential energy surface of the dihedral resembles the QM data more closely after optimization of the charges (**Fig. S1C, orange**).

Supplementary Figures

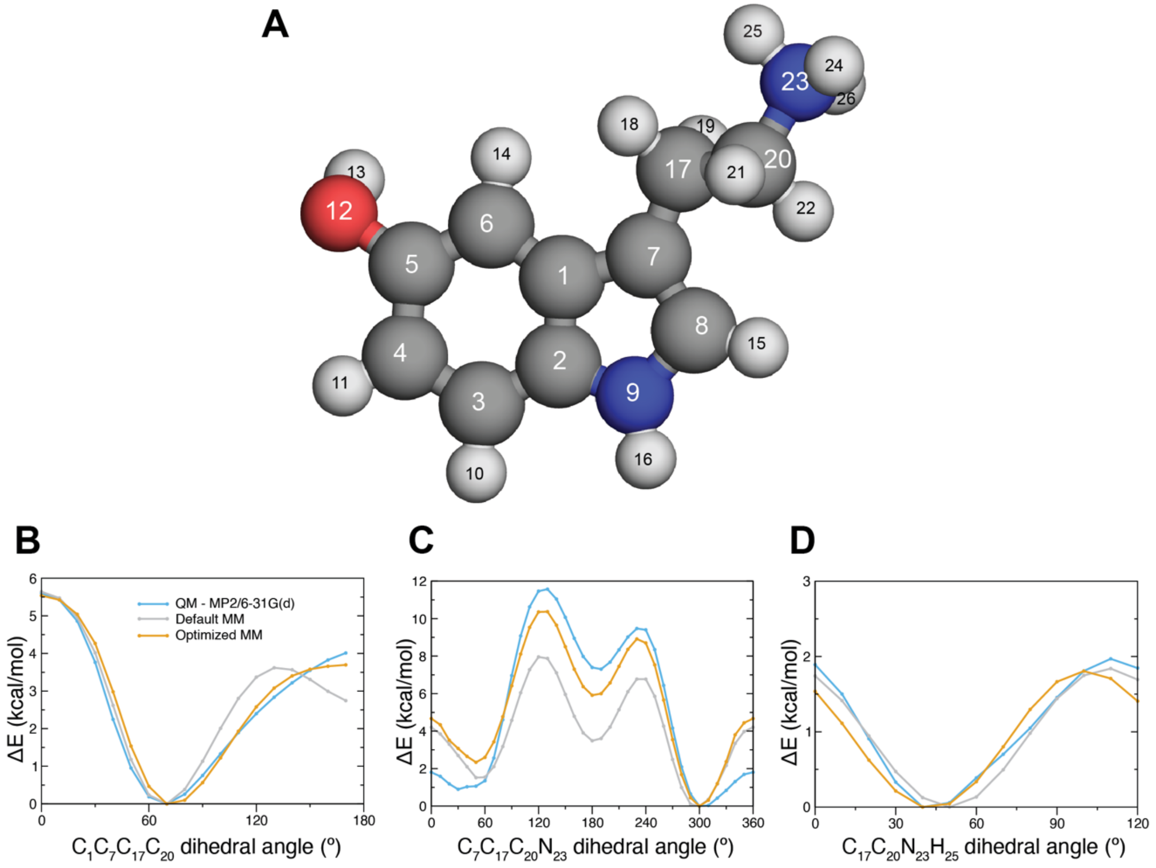

Figure S1. Parameterization of the 5-HT+ force field. **(A)** Atomic structure of 5-HT+, indicating atom labels used. **(B-D)** Potential energy curves calculated with MP2/6-31G(d) (blue) and with the default (gray) and optimized (orange) CGenFF, for various dihedrals and plotted as the energy relative to that of the equilibrium angle. The dihedral angles examined were: **(B)** the $C_1C_7C_{17}C_{20}$ dihedral angle between 0° and 180°, **(C)** the $C_7C_{17}C_{20}N_{23}$ dihedral angle between 0° and 360°, and **(D)** the $C_{17}C_{20}N_{23}H_{25}$ dihedral angle between 0° and 120°. Note that, the parameters of the torsions in $C_7C_{17}C_{20}N_{23}$ (C) and $C_{17}C_{20}N_{23}H_{25}$ (D) were kept unmodified; nevertheless, the optimized model produces a superior potential energy surface for $C_7C_{17}C_{20}N_{23}$ due to updated atomic charges.

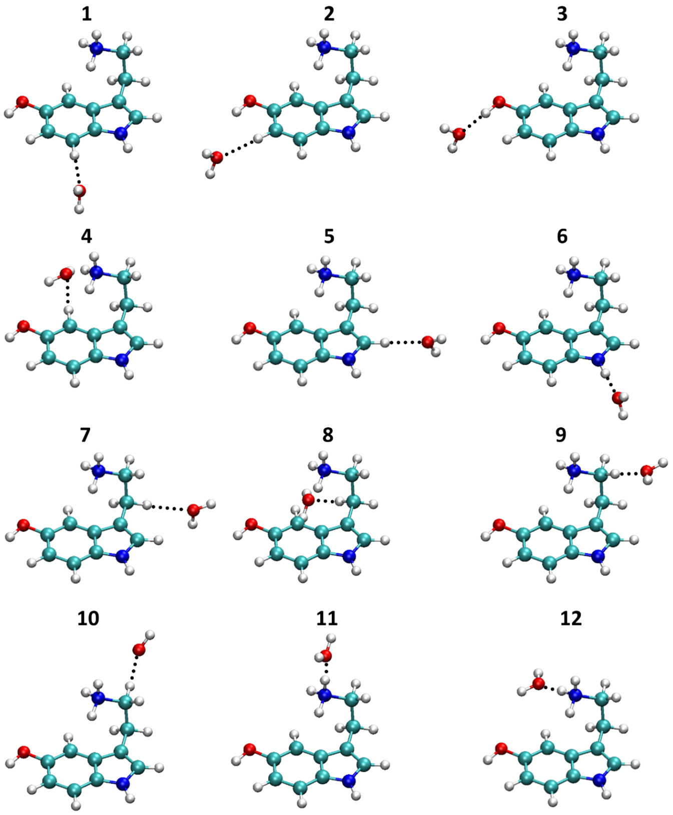

Figure S2. *Ab initio* optimized geometries of 5-HT$^+$-water complexes used in the calibration of an optimized molecular mechanics forcefield for 5-HT$^+$.

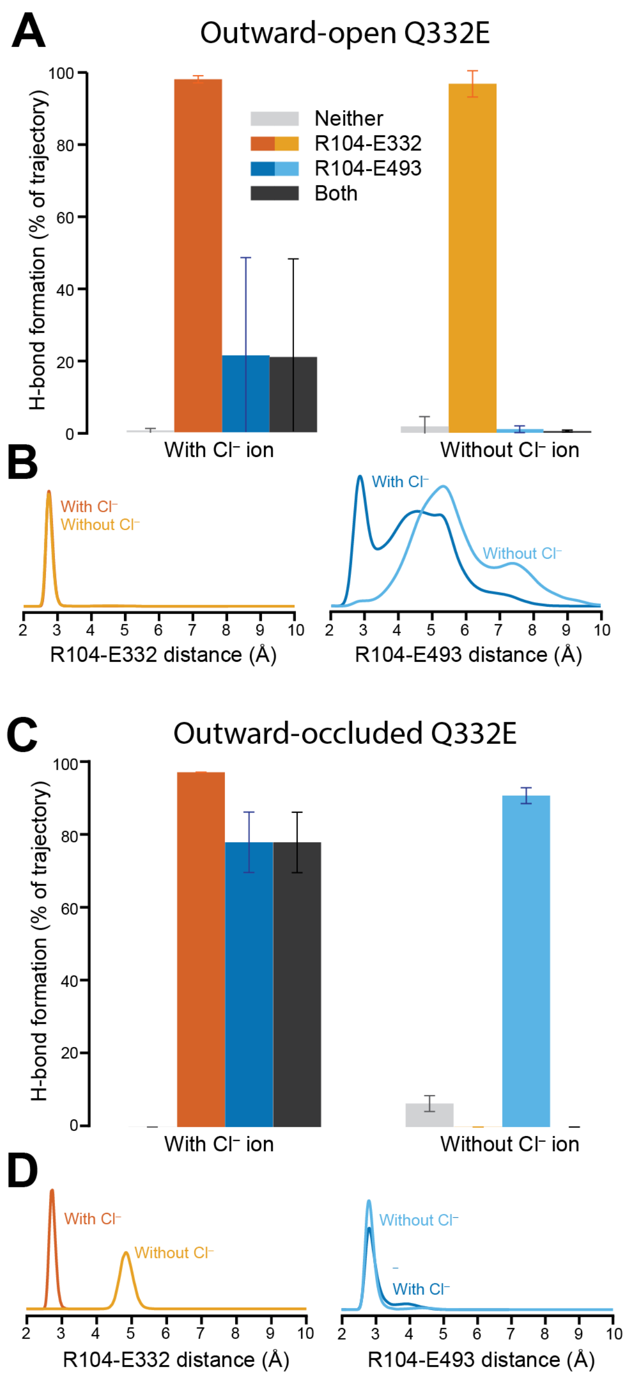

Figure S3. Behavior of the interaction network connecting the Cl^- binding site to the extracellular pathway salt bridge in the Q332E mutant of SERT. Simulations were initiated with **(A, B)** outward-open and **(C, D)** outward-occluded conformations for 0.5 μs simulations (n=4 or n=8, respectively). Substrate 5-HT^+ and two Na^+ ions were placed within their respective sites. **(A, C)** Formation of a hydrogen bond (distance <3.2 Å) between any donor atom of Arg104 and any acceptor atom of either the mutated Glu332 (*orange bars*), Glu493 (*blue bars*) or both (*black bars*), either in the presence (*left*) or absence (*right*) of a Cl^- ion initially placed at its reported binding site. Gray bars represent the fraction of time that neither H-bonds are formed. Error bars reflect standard deviations across repeat trajectories. In (**B, D**) the distance was measured either in the presence (*darker lines*) or the absence (*lighter lines*) of a Cl^- ion.

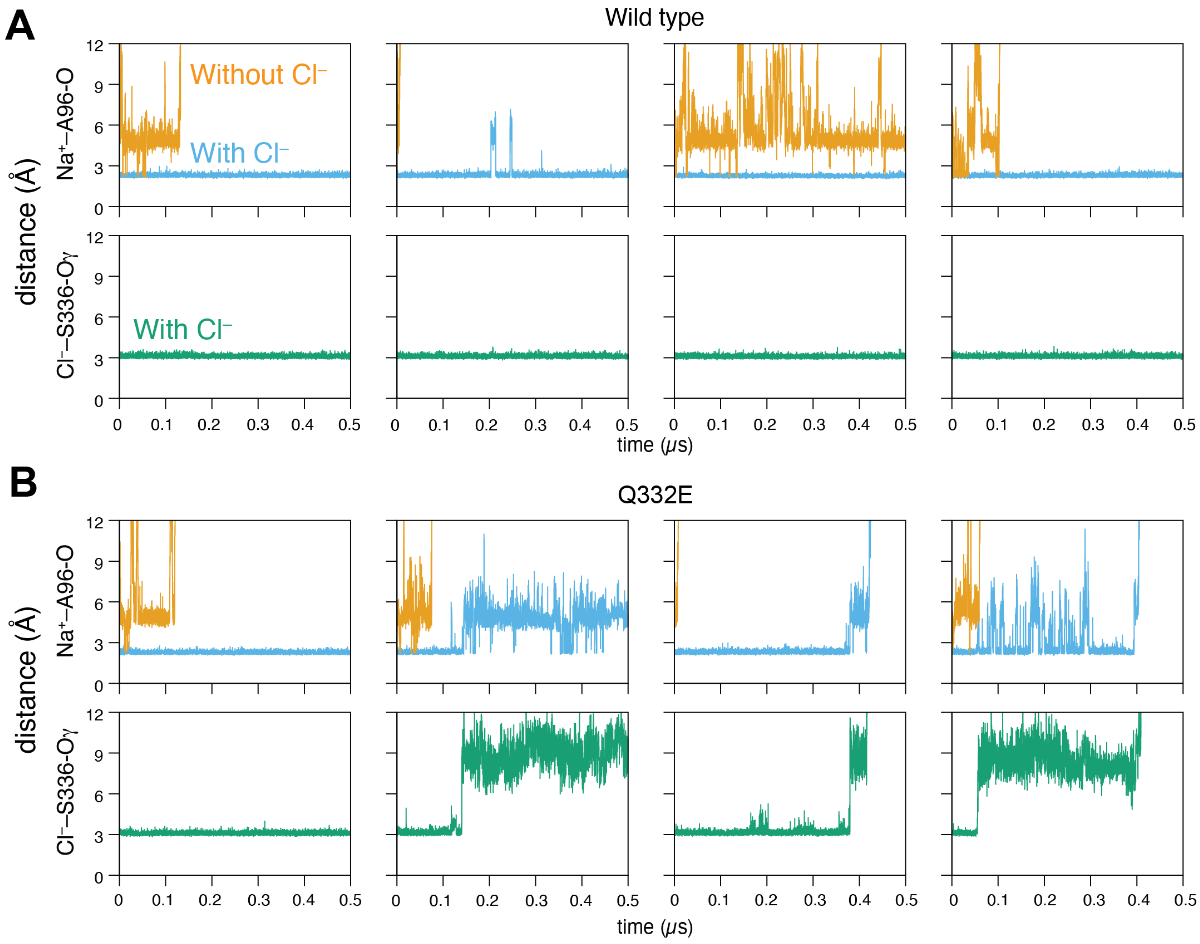

Figure S4. Interdependence of Cl^- and Na^+ occupancy in simulations of the outward-open conformation of SERT. Simulations of **(A)** wild type, and **(B)** mutant Q332E were carried out with or without Cl^- bound in at least four independent trajectories (columns). The distance of the Cl^- ion, when present, to the sidechain oxygen of Ser336 (green line), a representative of the chloride site, is compared to the distance of the Na^+ ion to the backbone oxygen of Ala96, a representative of the Na1 site, in simulations either with (cyan) or without (orange) the initially-bound Cl^- ion.

Protein	Sequence ID	Sequence Length	Start	TM10	End
hSERT SLC6A4	PDB 5I71	74-615	485	GAYVVKLLEEYAT-GPAVLTVALIEAVAVSWFY	516
hDAT SLC6A3	Q01959	1-620	468	GIYVFTLLDHFAA-GTSILFGVLIEAIGVAWFY	499
hNET SLC6A2	P23975	1-617	465	GIYVLTLLDTFAA-GTSILFAVLMEAIGVSWFY	496
hGAT2 SL6A13	Q9NSD5	1-602	439	GMYVFQLFDYYAASGMCLLFVAIFESLCVAWVY	471
hGAT3 SLC6A11	P48066	1-632	459	GMYIFQLFDSYAASGMCLLFVAIFECICIGWVY	491
hBGT1 SLC6A12	P48065	1-614	444	GMYIFQLFDYYASSGICLLFLSLFEVVCISWVY	476
hTauT SLC6A6	P31641	1-620	451	GMYVFQLFDYYAASGVCLLWVAFFECFVIAWIY	483
hCT1 SLC6A8	P48029	1-635	466	GMYVFQLFDYYSASGTTLLWQAFWECVVVAWVY	498
hGAT1 SLC6A1	P30531	1-599	443	GIYVFKLFDYYSASGMSLLFLVFFECVSISWFY	475
hGlyT2 SLC6A5	Q9Y345	1-797	625	GIYMFQLVDTYAA-SYALVIIAIFELVGISYVY	656
hPROT SLC6A7	Q99884	1-636	446	GMYWLVLLDDYSA-SFGLMVVVITTCLAVTRVY	477
hGlyT1 SLC6A9	P48067	1-706	520	GIYWLLLMDNYAA-SFSLVVISCIMCVAIMYIY	551
hATB0+ SLC6A14	Q9UN76	1-642	470	GIYWVHLIDHFCA-GWGILIAAILELVGIIWIY	501
hB0AT1 SLC6A19	Q695T7	1-634	478	GQYWLSLLDSYAG-SIPLLIIAFCEMFSVVYVY	509
hB0AT3 SLC6A18	Q96N87	1-628	464	GNYWLEIFDNFAA-SPNLLMLAFLEVVGVVYVY	495
hSIT1 SLC6A20	Q9NP91	1-592	453	GNYWFDIFNDYAA-TLSLLLIVLVETIAVCYVY	484
hB0AT2 SLC6A15	Q9H2J7	1-730	517	GNYFVTMFDDYSA-TLPLLIVVILENIAVCFVY	548
hNTT4 SLC6A17	Q9H1V8	1-727	516	GNYFVTMFDDYSA-TLPLTLIVILENIAVAWIY	547
hNTT5 SLC6A16	Q9GZN6	1-736	558	GSYFIRLLSDYWI-VFPIIVVVVFETMAVSWAY	589

Figure S5. Multiple Sequence Alignment of the 19 SLC6 transporters. The full UniProt sequences and SERT structure PDB ID 5I71 were aligned using Promals3D [Ref 31]. The figure shows the aligned residues of TM10, using SERT as a reference. Glu493 and Glu494 in SERT are highlighted together with the corresponding positions in the other SLC6 members in a purple box. Fully conserved residues are highlighted with orange background.

Supplementary Table 1. Properties of the two most stable 5-HT$^+$ conformers

	Dipole moment (D)		$\Delta E = E(1) - E(2)$, kcal/mol
	Conformer 1	Conformer 2	
MP2/6-31G(d)	13.48	7.91	7.3
Default CGenFF	14.76	9.44	3.6
Optimized CGenFF	12.01	7.86	5.7

Supplementary Table 2. Interaction H-bond distances (r, in Å) and interaction energies (E, in kcal/mol) for 5-HT$^+$-water complexes (***structures 1-12***). QM data are compared with MM values calculated with default CGenFF and with the optimized forcefield.

5-HT$^+$-H_2O Complex	HF/6-31G(d)		Default CGenFF		Optimized CGenFF	
	$r_{H...O}$, Å	*E (kcal/mol)*	$r_{H...O}$, Å	E (kcal/mol)	$r_{H...O}$, Å	E (kcal/mol)
1	2.33	-5.8	2.57	-4.5	2.53	-5.4
2	2.31	-6.1	2.51	-5.1	2.50	-6.1
3	1.90	-11.3	1.84	-10.0	1.82	-11.1
4	2.29	-11.0	2.22	-9.6	2.23	-10.5
5	2.26	-7.0	2.19	-7.0	2.21	-6.6
6	1.95	-10.5	1.84	-9.1	1.82	-10.2
7	2.33	-6.6	2.55	-6.0	2.58	-5.2
8	2.32	-7.8	2.51	-9.1	2.51	-8.0
9	2.26	-8.1	2.49	-8.1	2.51	-7.5
10	2.23	-8.5	2.48	-8.4	2.48	-8.1
11	1.84	-16.2	1.72	-18.3	1.72	-17.9
12	1.85	-17.2	1.73	-19.9	1.72	-19.0
